# Systems genetics approaches for understanding complex traits with relevance for human disease

**DOI:** 10.7554/eLife.91004

**Published:** 2023-11-14

**Authors:** Hooman Allayee, Charles R Farber, Marcus M Seldin, Evan Graehl Williams, David E James, Aldons J Lusis

**Affiliations:** 1 Departments of Population & Public Health Sciences, University of Southern California Los Angeles United States; 2 Biochemistry & Molecular Medicine, Keck School of Medicine, University of Southern California Los Angeles United States; 3 Center for Public Health Genomics, University of Virginia School of Medicine Charlottesville United States; 4 Departments of Biochemistry & Molecular Genetics, University of Virginia School of Medicine Charlottesville United States; 5 Public Health Sciences, University of Virginia School of Medicine Charlottesville United States; 6 Department of Biological Chemistry, University of California, Irvine Irvine United States; 7 Luxembourg Centre for Systems Biomedicine, University of Luxembourg Luxembourg Luxembourg; 8 School of Life and Environmental Sciences, University of Sydney Camperdown Australia; 9 Faculty of Medicine and Health, University of Sydney Camperdown Australia; 10 Charles Perkins Centre, University of Sydney Camperdown Australia; 11 Departments of Human Genetics, University of California, Los Angeles Los Angeles United States; 12 Medicine, University of California, Los Angeles Los Angeles United States; 13 Microbiology, Immunology, & Molecular Genetics, David Geffen School of Medicine of UCLA Los Angeles United States; Max Planck Institute for Biology Tübingen Germany; Max Planck Institute for Biology Tübingen Germany

**Keywords:** systems genetics, complex traits, omics, mouse models, human populations

## Abstract

Quantitative traits are often complex because of the contribution of many loci, with further complexity added by environmental factors. In medical research, systems genetics is a powerful approach for the study of complex traits, as it integrates intermediate phenotypes, such as RNA, protein, and metabolite levels, to understand molecular and physiological phenotypes linking discrete DNA sequence variation to complex clinical and physiological traits. The primary purpose of this review is to describe some of the resources and tools of systems genetics in humans and rodent models, so that researchers in many areas of biology and medicine can make use of the data.

## Introduction

Complex traits, including common diseases, result from the combined effects of multiple genetic variations together with environmental factors. Genome-wide association studies (GWAS) have revealed that most complex traits are highly heterogeneous and can involve contributions from hundreds of genetic variants, each explaining a tiny fraction of susceptibility. Although GWAS have been very successful in identifying thousands of causal genetic loci, the underlying mechanisms and interactions remain, in most cases, poorly understood. The regions identified in GWAS usually contain blocks of credible SNPs, and one challenge is to pinpoint the causal gene in that locus of interest. A further challenge is that the majority of fine-mapped SNPs are found in non-coding regions of the genome with no obvious link to protein function. It is often assumed that such SNPs will regulate transcription of a proximal gene but this can be difficult to prove and, in some cases, such as FTO (Claussnitzer et al., 2015), has proven not to be the case. A third challenge is to understand how the genetic variation perturbs the system to affect molecular, physiological, or clinical traits.

**Figure 1.**
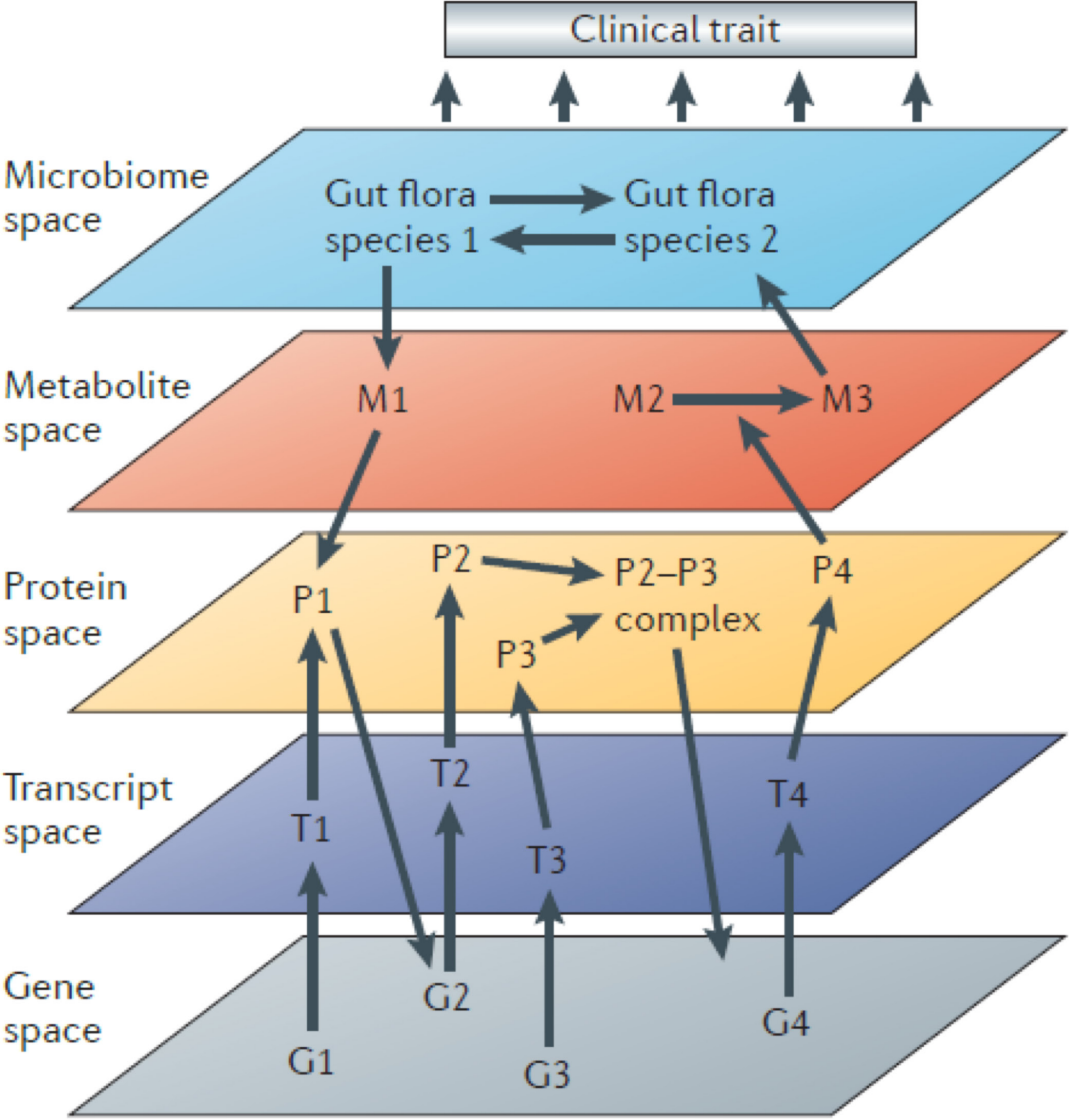
Systems genetics strategy for integration of clinical (and other complex) traits with molecular traits. In this cartoon, individuals in a cohort are examined for clinical or other complex traits of interest. Tissues from the same individuals are also examined using various omics technologies that quantitate to molecular traits. Genetic and environmental variations among the individuals will perturb the clinical and molecular traits. The relationships among the traits can then be statistically modelled using genetic mapping, correlation structure, causal inference, and network modelling. Figure adapted from Civelek and Lusis, 2014.

Systems genetics is an approach that seeks to understand the molecular and physiological phenotypes linking DNA sequence variation to complex clinical and physiological traits. It does this by using high-throughput ‘omics’ technologies to examine how intermediate molecular phenotypes, such as RNA, protein, and metabolite levels, are perturbed by natural genetic variation among individuals in the population and then attempting to relate these variations, using statistical methods, to physiological or clinical traits (Figure 1). Systems genetics approaches have proven very useful in identifying causal genes, pathways, and interactions underlying complex traits. We refer readers to a number of reviews of systems genetics strategies that cover historical developments, omics technologies, experimental designs, statistical analyses, and applications in detail (Ehrenreich et al., 2009; Civelek and Lusis, 2014 Baliga et al., 2017; Schughart and Williams, 2017; Seldin et al., 2019; Li and Auwerx, 2020; Molendijk and Parker, 2021).

The papers in this issue of eLife provide examples of its many applications across a broad spectrum of model systems. Vast amounts of systems genetics data (i.e., omics-level data collected in diverse populations of humans, mice, rats, flies, or yeast) are now compiled in publicly accessible databases or are available through collaborations. The primary purpose of this review is to describe some of the resources and tools of systems genetics, so that researchers in all areas of biology and medicine can make use of the data. Our focus is limited to studies in mammals although we note that many of the most important studies utilized yeast and flies. We begin with a brief overview, followed by descriptions of human and rodent tools and resources and end with thoughts about future directions.

### Overview of systems genetics

#### Design of systems genetics studies

A typical systems genetics study involves the following steps: (1) Identification of an important question, or set of questions, that could be addressed with a systems genetics study. (2) Selection of an appropriate cohort exhibiting variation in the traits of interest and of sufficient statistical power. (3) Phenotyping of the population for physiological, clinical, and omics traits of interest. (4) Integration of the resulting data through genetic mapping and statistical methods (Seldin et al., 2019). (5) Validation of hypotheses using methods such as gain/loss-of-function experimental studies. (6) For studies of disease, validation in human cohorts if possible. Developing a study de novo represents a substantial effort and cost, whether the study involves human or rodent populations. Thus, if possible, the study should be designed such that it can address many different questions and provide a resource for other researchers.

#### Human studies versus animal models

Complex traits such as common diseases typically involve not only many genes and environmental factors, but also complications such as lifestyle factors, aging-related changes, ethnic differences, sex differences, and in some cases gut microbiome interactions. It is important to design the study with these factors in mind. Several large human biomedical data and research resources have recently emerged, most notably UK Biobank. While such databases will play an invaluable role in future biomedical discoveries, they still currently possess at least two major caveats that are noteworthy. First, access to relevant tissues or organs may be limited and this limits systems genetics analyses. Second, the function of many genes is dramatically influenced by the environment but this is almost impossible to control and/or quantify in humans. For these and other reasons, studies of model systems such as genetically diverse cohorts of mice or rats have proven especially valuable for systems genetics. Notably, the ability to control the environment, age and sex of the animals, and other variables greatly reduce the number of individuals required for the study. As discussed below, a variety of useful mouse and rat population resources have been developed.

#### Flow of information

*S*ystems genetics can be used to follow the flow of information from DNA variation to intermediate phenotypes for the disease of interest. Thus, it is in some cases useful to interrogate the relevant tissues with different omics platforms (multi-omics) such as transcriptomics, proteomics, and metabolomics. Connecting the dots in this way, from DNA variation to molecular traits to the clinical trait, has been invaluable in identifying causal genes and relevant biological pathways. Information can, of course, also flow in reverse, and statistical methods can be useful in determining the direction of information flow (Figure 1).

#### Statistical analyses

Since information flow from DNA is unidirectional, causal pathways can be modeled and ‘mediators’ identified. For example, if both a clinical trait and the levels of a transcript are correlated and map to the same locus, one can condition on the transcript levels and ask whether a significant association between the locus and the clinical trait remains. If the association is eliminated, the results suggest that the effect on the clinical trait is not mediated by the transcript. Various causal inference tests have been developed and are typically referred to as mediation analysis (Schadt et al., 2005; Zhu et al., 2016). Mendelian randomization (MR) (Zhu et al., 2016) is one form of mediation analysis that has been particularly informative in dissecting causal influences between intermediate physiologic traits and disease pathophysiology in human studies. Higher-order interactions in biological systems are also conveniently modeled as network graphs using systems genetics data (Figure 1; Huang et al., 2018).

#### Reductionist versus systems genetics approaches

Biological research is now dominated by reductionist approaches, such as gain- or loss-of-function studies in mice. These approaches are powerful in that they establish causality, but they have some important limitations that hinder a more complete understanding of the architecture of complex traits, as discussed below. In contrast, systems genetics studies must generally be combined with experimental studies to conclusively establish causality. One constraint with the purely reductionist approach is that it usually involves perturbation of a single gene in a single genetic background and thus is unlikely to detect genetic interactions, such as modifier genes (Riordan and Nadeau, 2017). In other words, a genetic variation acts in the context of the genetic background, and by examining the effect of a gain or loss of function in only a single genetic background, an incomplete view of the function of the gene will be obtained. For example, engineered mutations in mice often exhibit strikingly different phenotypes when examined in different strains (Sittig et al., 2016).

Another important feature of systems genetics approaches is that they are relatively unbiased. Reductionist scientists usually generate hypotheses based on results from previous studies, and thus some genes or pathways are explored in great depth, whereas others are ignored. A recent study (Stoeger et al., 2018) found that more than one-quarter of coding genes have never been the subject of a single paper, whereas approximately 2000 genes (<10% of the coding genome) have dominated the literature. Systems genetics hypotheses, in contrast, are driven by natural variation paired with global measures of omics data and are therefore relatively unbiased. The power of natural variation derives from the multitude of genetic perturbations that occur in all combinations in a population (Heinz et al., 2013; Battle et al., 2017).

### Omics technologies

The different omics technologies used in systems genetics have been reviewed in detail (Hasin et al., 2017; Molendijk and Parker, 2021). In addition to genotyping, technologies have been developed that allow broad characterization of DNA modifications (epigenomics), gene expression (transcriptomics), protein expression (proteomics), small molecules (metabolomics), gut bacteria (metagenomics and metatranscriptomics). Below, we briefly review each of these technologies and their integration into systems genetics analyses of complex traits.

#### Epigenomics

In addition to variation in genomic sequence, chemical modifications to the nucleotide bases or DNA-bound histones, such as methylation, acetylation, and phosphorylation, can also vary between individuals (and cell types). Epigenomics is the study of such modifications and how they regulate gene expression (Allis and Jenuwein, 2016). The most well characterized of these modifications is the methylation of cytosine residues, which tend to cluster at repetitive CG dinucleotides in the proximal promoters of genes in regions referred to as CpG islands. Compared to unmethylated cytosines, methylated cytosine residues are protected from sodium bisulfite-induced deamination to uracil. Thus, it is possible to accurately quantitate the degree of methylation at individual cytosines by comparing the same DNA sequence before and after sodium bisulfite treatment.

Analogous to microarrays or SNP arrays, high-throughput methylation arrays have been developed that allow the simultaneous quantitative evaluation of >800,000 methylation sites throughout the genome. While such arrays do not cover all potential methylation sites, they have been designed to capture the proximal promoters of genes and other known important regulatory regions. Coupled with their relatively low cost, methylation arrays have allowed epigenome-wide association studies (EWAS) to be carried out on a large-scale (Campagna et al., 2021). In this approach, the degree of methylation at cytosines is tested for association with clinical traits or outcomes on a genome-wide level. Because of the large numbers of samples that have been archived or the ease with which they can be collected, most EWAS have been carried out with methylation levels in blood cell-derived DNA. Importantly, analytical methods have been developed that use the methylation data itself to deconvolute individual leukocyte populations (Houseman et al., 2012; Salas et al., 2022), which can be used as covariates in the analyses given the heterogeneity in blood cell number among individuals. Furthermore, databases that have curated EWAS datasets are publicly available (Li et al., 2019) and provide efficient access to published results for integration with other omics data, such as those described below.

An example of the application of systems genetics to understand epigenetic marks is the study by Orozco et al., 2015. The paper examined the contribution of DNA methylation to several complex traits relevant to heart disease, diabetes, and osteoporosis. DNA methylation in liver was examined in 90 mouse inbred strains from the Hybrid Mouse Diversity Panel (HMDP) (see below) using bisulfite sequencing. The DNA methylation levels in the liver were integrated with global transcript levels as well as proteomic and metabolomic data using correlation, mapping, and modeling. Many associations between epigenetic marks and clinical traits were identified, and causal inference tests were performed using the R statistical package CIT (Millstein et al., 2009). CIT performs a series of conditional probability tests to determine if the associations between a genetic locus and a trait are mediated by DNA methylation. About 25% of clinical trait associations were predicted to be causal. The study also revealed an example of how natural genetic variation can influence methylation levels. Mapping analysis of CpG methylation identified several hotspots in the genome in which a single locus was associated with variations in DNA methylation in many locations in the genome. One locus, on mouse chromosome 13, was associated with hundreds of CpGs. A candidate gene in the locus, methionine synthase reductase (*Mtrr*), was regulated in cis and its expression was highly correlated with methylation levels of CpGs mapping to the locus. A causal role of *Mtrr* in the methylation was demonstrated using genetically engineered mice (Orozco et al., 2015).

#### Transcriptomics

Most systems genetics studies performed to date have utilized transcriptomic data, largely because global gene expression can be measured at scale in a cost-effective manner using RNA sequencing (RNA-seq) (Stark et al., 2019). Additionally, most (>90%) disease associations identified by GWAS implicate non-coding variation, making the identification of trait-associated variation altering gene regulation (e.g., expression, splicing, etc.) a logical starting point to identify causal genes and mechanisms (Buniello et al., 2019). Although the original transcriptomic studies used gene expression arrays, the transcriptomic data used in contemporary systems genetics studies is almost exclusively generated by RNA-seq. There are different ‘flavors’ of RNA-seq analyses (e.g., total RNA-seq, mRNA-seq, small RNA-seq, etc.) but the general principle involves a multi-step process of RNA isolation from cells or tissues, conversion of RNA to cDNA, and next-generation sequencing. Gene expression is then quantified by aligning the resulting reads to a reference genome or transcriptome and counting the number of reads aligning to a particular gene, which equates to a digital readout of gene expression (Conesa et al., 2016).

One of the most useful systems genetics analyses involving transcriptomic data is the identification of expression quantitative trait loci (eQTLs) (Farber and Lusis, 2008). eQTLs are sets of genetic variants influencing gene expression. There are two types of eQTL, local (also referred to as *cis*-eQTL) and distant (also referred to as *trans*). Local eQTL are loci located in close proximity to the gene they regulate, whereas distant eQTL, as their name implies, are generally defined as being >~1 Mbp from the gene they regulate. Local eQTLs have been widely used to connect variants to genes in genetic studies. Another approach is transcriptome-wide association studies (TWASs) (Li and Ritchie, 2021). TWASs use eQTLs from a reference population to predict or impute gene expression in a much larger population. Imputed (genetically regulated) gene expression is then correlated with a complex trait to identify genes potentially driving disease.

RNA-seq is most commonly performed in heterogeneous tissues comprised of multiple cell types. However, genetic influences on gene regulation are often cell-type specific (Kim-Hellmuth et al., 2020). Recently, single-cell RNA-seq (scRNA-seq) has enabled the generation of complete transcriptomes from individual cells (Olsen and Baryawno, 2018). This provides the opportunity to study the genetics of gene regulation in individual cells or homogenous groups of cells. Generation of scRNA-seq data across a variety of organisms, tissues, and disease states has fueled an explosion of potential cell-specific contributions to complex outcomes. In a recent study, Chatterjee and colleagues created a *Drosophila* model which causes hyper-invasive multilayering of the follicular epithelia (Chatterjee et al., 2022). By comparing scRNA-seq profiles from ovaries of this cancerous strain to normal flies, the authors identified a link between *Keap1* and *Nrf2* as upstream components of tumorigenesis. However, scRNA-seq has technical limitations since it generally requires generating single-cell suspensions with fresh tissue. Therefore, scientists have also developed single-nuclei RNA-seq, which as the name implies involves sequencing RNA from nuclei of individual cells instead of whole-cell RNA. A variety of methods have been developed to analyze available scRNA-seq data to support or refute specific hypotheses. For example, a recent study developed Scallop, a statistical tool which enables quantification of transcriptional noise derived from scRNA-seq data (Ibañez-Solé et al., 2022). Here, the authors applied this method to normal or aged datasets in mice and humans to demonstrate that previously inferred transcriptional noise could be attributed to shifting cellular identities and thus not entirely stochastic. In another recent study, single-nuclei RNA-seq of brain tissue in 192 individuals identified 7607 genes with a local eQTL, 46% of which demonstrated cell-type-specific effects. The identified local eQTLs were found to colocalize with many loci identified in GWAS for Alzheimer’s disease, Parkinson’s disease, and schizophrenia, often in a cell-type-specific manner (Bryois et al., 2022). One caveat of scRNA analysis is the very low number of transcripts per gene in an average cell. Some estimates indicate that there are somewhere between 1 and 10 copies of RNA per gene per cell and this might be even lower in non-dividing cells (Eberwine et al., 2014; Mund et al., 2022). Hence, the accurate quantification using this method is likely restricted to abundant transcripts.

In the context of RNA-seq, the vast majority of transcriptomics data generated to date are based on short-read sequencing (reads of 75–150 bp). This has proven to be a powerful approach for quantifying expression at the level of individual genes. However, alternative splicing is a major gene regulatory mechanism that underlies disease states, including variation in disease-associated complex traits (Castaldi et al., 2022). While excellent for quantifying gene expression, short-read RNA-seq is not ideal for quantifying gene expression at the level of individual transcripts generated via alternative splicing. Long-read RNA-seq (reads of 10,000 bp or more) can address this limitation by enabling the identification and quantification of full-length transcripts. Long-read RNA-seq using technologies such as PacBio (Rhoads and Au, 2015) and Oxford Nanopore (Steinbock and Radenovic, 2015) promises to improve transcript quantification and our understanding of alternative splicing (Abood et al., 2023). Additionally, long-read RNA-seq coupled with short-read data in the short term should improve the identification and interpretation of genetically regulated alterations in splicing (i.e., splicing QTL) involved in disease (Abood et al., 2023). Ultimately, it is likely that, as the cost of generating long-read RNA-seq data continues to decrease, it will become the primary approach for characterizing and quantifying transcriptomes for systems genetics studies.

#### Proteomics

Advances over the past decade in sample preparation, fractionation methods, and mass spectrometry have led to the large-scale implementation of proteomics methods. Many studies now report quantitative tissue or cellular proteome measurements for >5000 proteins using minimalist sample preparation methods. Further, by using much deeper fractionation approaches, researchers can measure even more complete proteomes of 10,000–13,000 proteins in a single sample (Wang et al., 2019). These latter methods are not yet feasible on a larger scale due to the long run times and cost, but they presage a near future when scientists can measure the proteome as comprehensively and affordably as nucleotide sequencing has made transcriptomics. Several systems genetics studies have already utilized tissue proteomics to great affect and this approach is becoming a valuable part of the systems genetics armamentarium (Mitok et al., 2018; Parker et al., 2019; Xiao et al., 2022; Yu et al., 2023).

Other technologies capable of broadly surveying protein levels include the SOMAscan aptamer-based multiplexed platform based on the development of single-stranded DNA aptamers that show high affinity for protein targets (Gold et al., 2010) and the proximity extension assay developed by Olink (Assarsson et al., 2014). However, these methods are most commonly used on human plasma samples and their application to tissue samples is yet to be realized. The latest platform can now measure over 5000 proteins in a sample and has been used to consistently quantify plasma proteins from tens of thousands of human subjects. This method quantifies proteins when two antibodies to which barcodes are attached bind to the same protein, thus bringing the barcodes into close proximity with each other and allowing nucleic acid-based amplification. There are other promising techniques under development as well, including single-cell proteomics (Molendijk and Parker, 2021).

Similar to genetic studies of transcript levels, genetic variations affecting proteins can be classified into those in which the locus maps near the coding gene or those that map elsewhere in the genome. The former are termed ‘local’ protein QTL (pQTL) and presumably act in cis, affecting only the expression of the gene on the contiguous DNA strand. The latter are termed *trans* pQTL. The correlation between transcript and protein levels in genetic studies tends to be relatively modest (Liu et al., 2016).Thus, the information derived from a proteomics analysis will be quite different from that of a transcriptomics systems genetics study. Likewise, genetic loci affecting protein levels are often distinct from those that regulate the corresponding mRNA, even for variants acting in cis (Williams et al., 2022), for several reasons. First, the turnover of individual proteins varies from minutes to many days in cells; for instance, mitochondrial proteins exhibit particularly long half-lives (Minard et al., 2016). Moreover, protein turnover is under intense control by various post-translational modifications such as ubiquitination, phosphorylation, or acetylation. This is likely to be a major contributor to the non-linear relationship between mRNA and protein expression in cells. Second, many proteins form multi-protein complexes and this often leads to complex co-regulation of all the proteins that exist in such complexes. The archetypal example of this is the T cell receptor, which comprises six subunits. In T cells, surface expression of this receptor requires efficient expression and assembly of all six subunits in stoichiometric amounts and failure to make one single subunit results in rapid degradation of the remaining subunits (Weissman, 1994). Third, protein levels and functions can be affected by missense and nonsense variations that affect translation or processing. Thus, the genetic control of cellular protein levels is highly complex and requires more detailed analyses. Proteomics analyses are also capable of examining certain protein modifications that may be of functional relevance. Protein phosphorylation, in particular, is of great importance in intracellular signaling (Needham et al., 2022). In addition, other protein modifications have recently been quantified in systems genetics reference panels, such as UFMylation, which was demonstrated to impact muscle physiology (Molendijk et al., 2022).

#### Metabolomics

Similar to proteomics, high-throughput technologies have been applied to the characterization of the metabolome, often referred to as the pool of metabolites in biological systems, such as cells or tissues (Wishart, 2019). Two of the most commonly used methods include MS, coupled with either LC or gas chromatography (GC), or nuclear magnetic resonance (NMR). When done in an unbiased fashion, LC/GC-MS can detect and quantify thousands of small molecules in a particular sample. However, this approach is limited by not being able to provide absolute quantification of the features detected unless known standards are spiked into the sample being analyzed. By comparison, NMR-based metabolomics approaches can provide absolute levels with minimal or no pre-processing of samples, thus increasing efficiency, reducing time, and decreasing costs. However, NMR is typically not as sensitive as MS methods. Both metabolomics approaches have been applied in thousands of subjects from numerous human cohorts and in relation to multiple common diseases (Wishart, 2019). By being distal to mRNA and proteins but proximal to disease outcomes, metabolomics also offers the advantage of providing a level of biological data situated between DNA sequence variation and clinical traits.

Aside from characterizing the repertoire of small molecules, the coupling of metabolomics with genetic data has also allowed large-scale GWAS to be carried out simultaneously for thousands of metabolites, analogous to eQTL and pQTL analyses (Kastenmüller et al., 2015). Such metabolomic QTL (mQTL) have been very informative by revealing genetic determinants of small molecules derived from cellular and physiological processes and have provided insight into the intermediate mechanisms that could underlie association of loci with disease traits. mQTL summary statistics are also publicly available for download similar to other omics data through searchable web-based resources, thus facilitating efficient systems genetics analyses.

#### Gut microbiome

Systems genetics can also be applied to the gut microbiota. The various microbes can be identified and quantitated using shotgun sequencing of microbial DNA or sequencing of informative amplicons of the bacterial 16S gene. Whole microbial DNA sequencing frequently permits reads to be specifically assigned to individual species or genera. In contrast, 16S sequencing is much less expensive, but it generates data which are often analyzed at a much broader scale, for example at the phylum level. Gut microbe compositions are determined by the diet, host genetics, and other characteristics of the host such as aging. Usually, in humans, bacterial DNA is isolated from stool samples, whereas in mice, cecal contents or fecal pellets can be used. Large-scale population studies of gut microbes, which include fungi as well as bacteria, have been performed in an effort to relate gut bacterial composition to clinical outcomes or other traits (Kurilshikov et al., 2021). Because there are thousands of species in a single individual’s gut microbiome, and because the microbes exist as communities with other species, it has been difficult to identify specific relationships, although broad categories, such as the ratio of Firmicutes to Bacteroidetes phyla, have been related to traits such as obesity. Such studies are simplified in animal models, such as the mouse. For example, in a systems genetics study of microbes in a diverse set of ~100 inbred strains of mice, the species *Roseburia intestinalis* was found to be associated with resistance to atherosclerosis. Further studies with germ-free mice confirmed this and identified butyrate, a metabolite produced by *Roseburia*, as the intermediate (Kasahara et al., 2018). In this regard, analysis of bacteria-derived metabolites has been particularly informative (Jie et al., 2017). For example, Hazen and colleagues identified the metabolite trimethylamine-*N*-oxide, derived from dietary choline and carnitine, as a very significant cause of atherosclerosis (Tang et al., 2019). Systems genetics approaches have also successfully identified some host genetic loci with large effects on microbe compositions. The most significant of these is the lactase locus, which regulates the ability to hydrolyze dietary lactose. If an individual is deficient in the intestinal enzyme, the availability of lactose in the gut will lead to an increased abundance of bacteria that can utilize this sugar as an energy source.

### Population resources

#### Mouse and rat populations

The mouse and rat have played critical roles in systems genetics studies of complex traits. The popularity of rodents for systems genetics studies is due to many reasons, including the similarity between rodent and human physiology, the relatively lower cost compared to human studies, access to critical tissues and disease phenotypes, and the availability of populations designed for genetic mapping. In particular, rodent populations are critical for systems genetics studies investigating clinically relevant conditions (e.g., genetic × diet effects in obesity [Parks et al., 2013] or biomechanical bone strength [AlBarghouthi and Farber, 2019]) that are difficult or impossible to study in humans.

There are two types of rodent populations that are commonly used for systems genetics studies - genetic reference populations (GRPs) and outbred stocks. GRPs can be recombinant inbred lines derived from two or more inbred strains (Williams and Williams, 2017) or collections of inbred strains, such as the HMDP (Bennett et al., 2010) and the Hybrid Rat Diversity Panel (HRDP) (Tabakoff et al., 2019). Outbred stocks can begin from a variety of starting points, including initial crosses between inbred founder strains, with the resulting progeny forming a randomly mating population. The most commonly used genetic reference populations and outbred populations are listed in Table 1.

**Table 1.** Commonly used genetic reference populations (GRPs) and outbred populations in mice and rats.

GRPs	Species	Inbred or outbred	# of strains	Description	Data repository
BXD	Mouse	Inbred	140	Recombinant inbred lines generated from C57BL/6J and DBA/2J founders	https://genenetwork.org/
HMDP	Mouse	Inbred	~100	A set of ~100 classical laboratory inbred strains and multiple recombinant inbred line panels	http://systems.genetics.ucla.edu
CC	Mouse	Inbred	~50–75	A panel of ~75 recombinant inbred lines derived from eight genetically diverse inbred founders	http://csbio.unc.edu/CCstatus/index.py
HRDP	Rat	Inbred	99	A set of ~100 classical laboratory inbred strains and recombinant inbred line panels	http://phenogen.org https://genenetwork.org/
HXB/BXH	Rat	Inbred	30	Recombinant inbred lines generated from the spontaneously hypertensive rat (SHR/OlaIpcv) and Brown Norway (BN.Lx/Cub) founders	http://phenogen.org https://genenetwork.org/
DO	Mouse	Outbred		An outbred population derived from eight genetically diverse inbred founders	https://genenetwork.org/

The two different populations have unique advantages and disadvantages (Jurrjens et al., 2023). The main advantage of GRPs is that animals within each strain in the population are identical and replicable. This eliminates the need for costly genotyping for each new study and provides a stable set of genotypes on which phenotype data can be accumulated. GRPs are ideal for investigating gene × environment (GXE) interactions given that phenotypes can be measured in strains exposed to different environmental conditions. They are also useful for the genetic analysis of traits with low heritability since having replicates of genetically identical individuals from the same strain decreases noise and increases heritability (Bennett et al., 2010; Keele, 2023). Heritability estimates provide a valuable metric to quantify the genetic and/or environmental contributions to complex outcomes. However, these have been difficult to accurately assess in humans, and studies have suggested differences in heritability estimates depending on methodological approaches used (Mayhew and Meyre, 2017). Several improvements to human heritability estimates have been proposed such as gene network-based approaches which have the power to inform context-specific estimates of complex traits (Feng et al., 2022). However, one disadvantage for rodent studies is that most GRPs consist of a relatively small number of strains (~25–140), limiting statistical power to detect subtle genetic effects on clinical and molecular traits.

By comparison, outbred stocks are not limited by sample size, enabling the detection of more subtle genetic effects and relationships among traits. This additional power can be important when exploring the polygenicity of complex traits. Additionally, outbred stocks accumulate recombinations at every generation, increasing mapping resolution over time, as compared to GRPs which have a fixed set of recombinations and, thus, fixed mapping precision. The main disadvantage of outbred stocks is the uniqueness of each animal, which requires de novo genotyping. This limits the number of disease phenotypes that can be captured on the same set of mice. Also, while GXE interactions can be studied in outbred stocks, it is much more straightforward with GRPs. Below, we discuss the most popular GRPs and outbred stocks used in both mice and rats. We note that, while not reviewed here, advanced intercross lines have also been extensively used to map complex traits. For example, a recent intercross between inbred QSi5 and 129T2/SvEms mouse lines identified 37 new QTLs which associated with cardiac interatrial septation (Moradi Marjaneh et al., 2023).

#### Widely used GRPs and outbred stocks

##### BXD

The BXD GRP is a set of recombinant inbred strains derived from crosses between the C57BL/6J and DBA/2J founders. The initial set of BXD strains was developed in the early 1970s by Dr. Benjamin Taylor at the Jackson Laboratory (Taylor et al., 1973). This set was subsequently expanded to approximately 140 strains (Peirce et al., 2004; Ashbrook et al., 2021). As stated above, one of the main advantages of inbred GRPs is the ability to accumulate data on the same genotypes over time. The BXD GRP has been used to map a wide range of complex traits (reviewed in Ashbrook et al., 2021). As an example, a recent study took advantage of the accumulation of de novo mutations over 50 years in the BXD to identify alleles in *Mutyh*, a known driver of colorectal cancer, that modulate specific germline mutagenesis signatures (Sasani et al., 2022). The BXDs are one of the most well-characterized GRP in any species. To date, over 7500 phenotypes, in disease categories such as cardiovascular disease (CAD), behavior, addiction, and cancer, just to name a few, have been collected. Additionally, over 100 ‘-omics’ (transcriptome, metabolome, DNA methylation, and proteome) datasets on multiple tissues have been generated in the BXD GRP, making it a powerful systems genetics resource. A recent integrative systems genetics study of bile acid metabolism in the BXD used different diets, bile acid profiling, liver transcriptomics, and metabolic phenotyping to identify *Ces1c* as a master regulator of plasma tauroursodeoxycholic acid, a bile acid known to have health-promoting actions (Li et al., 2022). All of these data are stored on the GeneNetwork2 database (https://genenetwork.org/) and can be used for interactive systems genetics studies.

##### HMDP

The HMDP is a GRP comprised of a set of commercially available recombinant inbred strains (including the BXD strains discussed above) and classical laboratory strains (Bennett et al., 2010). Most experiments using the HMDP have included approximately 100 strains, though the number of strains that could be used is larger. The HMDP was designed to use available resources to address two issues, the limited statistical power of most strain panels and the lower mapping resolution of RILs. In the HMDP, recombinant inbred strains increase sample size and power, while the small haplotype blocks found in classical inbred strains increase mapping resolution. The HMDP has been used to study a wide range of systems genetics studies of disease phenotypes such as plasma lipids (Bennett et al., 2010), obesity (Parks et al., 2013), bone mass (Farber et al., 2011), fatty liver disease (Hui et al., 2015; Hui et al., 2018), atherosclerosis (Bennett et al., 2015), heart failure (Rau et al., 2015), cellular phenotypes (Davis et al., 2013; Zhou et al., 2015), and various omics datasets (Ghazalpour et al., 2011; Ghazalpour et al., 2014; Hartiala et al., 2014; Orozco et al., 2015). Furthermore, the HMDP is ideal for GXE experiments and has been used to identify loci and genes responsible for differences in obesity and atherosclerosis between chow and high-fat diets and response to environmental exposures (Lavinsky et al., 2016; Maazi et al., 2019; Tuominen et al., 2021). Recently, the HMDP was also used to identify coagulation factor XI as an endocrine factor produced in the liver impacting cardiac fibrosis, inflammation, and heart failure (Cao et al., 2022). Extensive genetic, phenotypic, and omics (transcriptomic and proteomic) data collected in the HMDP can be found at https://systems.genetics.ucla.edu/HMDP/.

##### CC and DO mice

The collaborative cross (CC) and diversity outbred (DO) mice are multi-parental populations created by ‘combining’ eight genetically diverse inbred founders: A/J, C57BL/6J, 129S1/SvImJ, NZO/H1LtJ, NOD/LtJ, WSB/EiJ, PWK/PhJ, and CAST/EiJ (Churchill et al., 2004; Churchill et al., 2012; Svenson et al., 2012; Saul et al., 2019; Solberg Woods and Palmer, 2019). After the founders were intercrossed in a balanced design, the resulting progeny were used to create a set of recombinant inbred lines (CC) or randomly bred to create DO mice. The genomes of the CC/DO founders have been sequenced and from these data we know that at least ~45 million SNPs and INDELs/structural variants are segregating in the CC and DO mice (Keane et al., 2011). This level of variation is significantly higher than existing mouse GRPs (e.g., ~5 million variants are segregating in the BXD created from classical laboratory strains [Wang et al., 2016]) and this level of variation is more than the common variation seen across diverse human populations.

The original goal for the CC was the generation of 1000 recombinant inbred strains (Churchill et al., 2004); however, genetic incompatibilities among the founders caused most strains to be lost during development. Today there are roughly 50–70 strains that are accessible in the United States. The relatively small number of strains and the increased genetic diversity (which reduces statistical power to identify individual effects) has limited the use of the CC for genetic mapping of complex traits.

The DO has all of the advantages and disadvantages of outbred stocks that are described above. A consideration when designing systems genetics studies using the DO is the increased sample size required over crosses between classical strains to identify QTL in the DO due to the increased genetic variation (Svenson et al., 2012). Despite this, the DO provides a powerful platform to integrate polygenic traits and has been used to perform GWAS and systems genetics studies for stem cell biology (Skelly et al., 2020; Aydin et al., 2023), insulin secretion (Keller et al., 2019), and a wide variety of other disease-associated traits (Chick et al., 2016; AlBarghouthi et al., 2021; Xiao et al., 2022).

##### HXB/BXH rats

The HXB/BXH GRP is a set of 30 RILs generated by intercrossing the spontaneously hypertensive rat with the normotensive Brown Norway rat strain (Pravenec et al., 1989; Pravenec et al., 2014). The HXB/BXH GRP has been used for genetic and systems genetic studies of blood pressure (Pravenec et al., 1989), alcohol-related traits (Vanderlinden et al., 2014; Saba et al., 2015; Lusk et al., 2018), metabolic and cardiovascular traits (Morrissey et al., 2011; Pravenec et al., 2018), among others. The HXB/BXH GRP was recently used to identify a pleiotropic QTL affecting hippocampal neurogenesis and serum glucose (Senko et al., 2022). Using a systems genetics approach integrating transcriptomics data the Telo2-interacting protein 2 (Tti2) gene was identified as a strong candidate and subsequently validated using a rat Tti2 knockout.

##### HRDP

The HRDP is a rat GRP similar to the HMDP. Like the HMDP, the HRDP is a panel consisting of both RILs and classical inbred strains, with the goal of maximizing power and mapping resolution using existing resources. Specifically, the HRDP is comprised of 35 inbred strains, 30 strains from the HXB/BXH RIL, and 34 strains from the LEXF/FXLE RIL (Voigt et al., 2008; Tabakoff et al., 2019). Simulations with the HRDP has suggested that an experiment with the full panel has 80% power to detect QTL accounting for approximately 12% of the variance for complex traits (Tabakoff et al., 2019). While few studies have mapped QTL in the HRDP, mapping resolution should be quite good as the GRP has a median haplotype block in of approximately 150 kbp (Tabakoff et al., 2019).

### Human populations

Large-scale GWAS, since their first implementation 20 years ago, have been instrumental in elucidating the genetic architecture of complex traits. More recently, this approach has been expanded to include meta-analyses using data from multiple datasets and measurement types (e.g., phenome-wide association studies). This has led the international scientific community to build on this success and develop multi-ancestry biobanks with tens and hundreds of thousands of subjects for systems genetics studies that leverage the vast amount of clinical data and archived blood and tissue samples available in healthcare systems around the world. Some of the datasets provide the advantage of including individuals of non-European ancestry and can be used to determine whether epidemiological and genetic findings are applicable across ethnicities. Importantly, this addresses one criticism of human genetic studies that subjects of European ancestry have been overrepresented in GWAS. Furthermore, a collaborative network comprised of 24 international biobanks has been formed, termed the Global Biobank Meta-analysis Initiative (GBMI), with the express goal of facilitating large-scale genetic analyses of selected common diseases (https://www.globalbiobankmeta.org/; Zhou et al., 2022). Thus far, over 2.2 million genotyped multi-ancestry subjects from both population-based and hospital/health center-based cohorts have been included in the GBMI, which will undoubtedly increase as more groups join this initiative. While a description of every available biobank is beyond the scope of this review, below we highlight some of those that have extensive resources available and have been used widely (Table 2).

**Table 2.** Commonly used human biobanks for epidemiological and genetics studies.

Cohort	Ancestry (N)	Disease traits	Biomarkers	Genomics	Transcriptomics	Proteomics	Metabolomics	Data repository
UK Biobank	European, Asian, African, Other (502,492)	✓	✓	✓		✓	✓	https://www.ukbiobank.ac.uk/
FinnGEN	European (500,000*)	✓		✓				https://www.finngen.fi/en
Biobank Japan	Asian (260,000)	✓	✓	✓		✓	✓	https://biobankjp.org/en/
China Kadoorie Biobank	Asian (512,000)	✓	✓	✓		✓	✓	https://www.ckbiobank.org/
TOPMed	European, African, Hispanic, Asian (205,092)	✓	✓	✓	✓	✓	✓	https://topmed.nhlbi.nih.gov/
BioVU	European (300,000^†^)	✓	✓	✓				https://victr.vumc.org/biovu-description/
Millions Veteran Program	European, African, Hispanic, Asian (950,000^†^)	✓	✓	✓				https://www.mvp.va.gov/pwa/
Geisinger MyCode	European (300,000^†^)	✓		✓				https://www.geisinger.org/precision-health/mycode

* Indicates goal of subject recruitment.

† Recruitment still ongoing. Citations for the studies are provided in the text.

#### The Genotype-Tissue Expression (GTEx) project (https://gtexportal.org/home/)

The GTEx project consists of >500 postmortem samples from ~50 tissues in humans. The project was initiated with the goal of providing a resource of organs-specific actions of eQTL variants (Consortium, 2013) where intersection with common disease association SNPs could implicate or disregard a specific tissue of action. These data have been particularly useful in providing researchers with a fairly comprehensive paired tissue RNA-seq and genome datasets among the same individuals. More recent efforts have expanded this resource to encompass measures of chromatin accessibility (Zhang et al., 2021a), long-read RNA-seq (Glinos et al., 2022), and single-nuclear RNA-seq (Jones et al., 2022a) in the same individuals.

#### The UK Biobank

The UK Biobank is a large, multi-site cohort that recruited a total of 503,325 individuals in the UK between 2006 and 2010 (Bycroft et al., 2018). Data and health-related information for ~6700 phenotypes are available on each participant, including biological measurements, lifestyle indicators, environmental exposures, imaging, blood and urinary biomarkers, metabolomics, proteomics, and genetic variants and exome and whole-genome sequencing. Since making the data available through an application-based approval process, hundreds of research groups have published thousands of studies using the UK Biobank cohort. Of all biobanks, access to primary level data in UK Biobank is least restrictive and can be obtained through a proposal and application review process.

#### FinnGen

The goal of the FinnGen biobank is to recruit ~500,000 subjects drawn from epidemiological and disease-based cohorts as well as hospital biobank samples in Finland (Tabassum et al., 2019). These data will be combined with registry data that record healthcare events over the entire lifespan. As of December 2022, over 356,000 participants have been recruited and genotyped on SNP arrays and the goal is to finish recruitment by the end of 2023. Results of GWAS analyses for over 2200 disease phenotypes are made publicly available twice year, which can be leveraged for meta-analyses and investigation of candidate loci/genes. However, plans to extend the scope of FinnGen to characterization of biomarkers and other omics data in participants have not been announced.

#### Biobank Japan

Biobank Japan is a national biobank of ~260,000 patients who were enrolled at 12 hospitals throughout Japan between 2003 and 2008 (Hirata et al., 2017; Nagai et al., 2017). The Biobank Japan study examines 51 common diseases in its participants and whole-genome SNP array data is already available in over 215,000 subjects, with plans to extend genetic analysis to exome and whole-genome sequencing. Other omics data, such as metabolomics and proteomics in serum, has also been collected on a subset of the cohort. Similar to UK Biobank and FinnGen, summary statistics are publicly available and can be leveraged for various systems genetics analyses.

#### China Kadoorie Biobank

The China Kadoorie Biobank has recruited over 500,000 adults from 10 geographically diverse (urban and rural) areas of China from 2004 to 2008 (Chen et al., 2011). Extensive data was collected at baseline but the China Kadoorie Biobank is also focused on prospective data collection with surveys of participants carried out every 4–5 years. In addition to genetic (whole-genome genotyping and sequencing) and disease outcome data, biological samples have been collected for biomarker, metabolomics, and proteomics analyses.

#### TOPMed

The Trans-Omics for Precision Medicine (TOPMed) program is an NIH-sponsored precision medicine initiative consisting of over 200,000 participants drawn from over 85 cohorts. The primary goal is to integrate clinical, genetic (whole-genome sequencing), and other omics data (e.g., epigenomics, transcriptomics, proteomics, and metabolomics) to gain a better understanding of heart, lung, blood, and sleep disorders (Brody et al., 2017). One notable advantage of TOPMed is the broad diversity in participants’ ancestries, including those of African, Hispanic/Latino, and Asian backgrounds. However, access to individual-level data and summary statistics is more restrictive than, for example, UK Biobank but can be gained through an application review process.

#### BioVU

BioVU is an electronic health record (EHR)-based biobank in which over 300,000 participants have been recruited at Vanderbilt University (Roden et al., 2008). It is one of the largest single-site biobanks and also leverages whole-genome genotyping, EHR data, and archived plasma samples to investigate the genetic basis of thousands of disease phenotypes. However, access to the data is also more restrictive than some other biobanks and requires being a Vanderbilt University faculty member. BioVU is also part of the large Electronic Medical Records and Genomics (eMERGE) national network of cohorts and biobanks (https://emerge-network.org/), which is organized and funded by the NIH to carry out large-scale, high-throughput genetic research with EHR-based data.

#### MVPMillion Veteran Program

The Million Veteran Program (MVP) is a national research program organized and sponsored by the Veterans Administration Office of Research and Development. The goals of the MVP are to better understand the interrelationships between health outcomes, genetic determinants, behavioral traits, and environmental factors, with a particular focus on improving the care of US Veterans (Gaziano et al., 2016). It is one of the largest biobanks and, as of mid 2023, has recruited over 950,000 subjects of different ancestries who are representative of the Veteran population. Thus, the MVP represents one of the largest research programs in the world studying genes and health outcomes.

#### Geisinger

The Geisinger MyCode Initiative is another EMR-based biobank that is enrolling participants from a rural, integrated health system serving central and northeastern Pennsylvania (Carey et al., 2016; Jones et al., 2022b). SNP genotyping and exome sequencing is being carried out on all participants with the same overall goal as other biobanks of discovering novel genetic associations with disease and therapeutic targets. However, one unique distinction between Geisinger MyCode and other biobanks is the decision to return clinically actionable genetic findings (e.g., BRCA1 mutation status) back to subjects. This is based on surveys of participants who generally indicated they are in favor of receiving such information (Schwartz et al., 2018).

### Analytic approaches

#### Correlation structure and causal modeling

Correlation is a simple procedure for relating molecular and clinical/physiological traits. Of course, the traits can be correlated for multiple reasons. However, since information from DNA is unidirectional, causal pathways can be modeled. For example, if a clinical trait and the levels of a transcript are correlated and map to the same locus, researchers can condition on the transcript levels and ask whether a significant association between the locus and the clinical trait remains. If so, the results suggest that the effect on the clinical trait is not mediated by the transcript. Furthermore, SNPs are often associated with multiple clinical traits, which can be due to either pleiotropy, direct or indirect genetic effects, or environmental/non-genetic factors. Mediation analysis can therefore be used to quantify the proportion of direct and indirect effects of the exposure on the outcome of interest via the intermediate biomarker as the mediator. One straightforward application of this approach would be to use causal mediation analysis to determine whether association of a variant with insulin resistance is mediated through an effect on BMI. MR (see below) is another form of mediation analysis that has especially strict criteria in that the mediator (such as the levels of a protein) is required to explain all of the association between an SNP and a complex trait. Various forms of causal inference tests have been developed for mediation analysis (Schadt et al., 2005; Zhu et al., 2016; Zeng et al., 2021; Crouse et al., 2022).

#### Mendelian randomization

The gold standard for determining causality with biomarkers has relied on randomized clinical interventions. However, MR has emerged as an alternative and highly efficient strategy for leveraging genetic data to make causal inferences (Smith and Ebrahim, 2003; Didelez and Sheehan, 2007; Lawlor et al., 2008; Burgess et al., 2017). In this approach, MR uses Mendel’s laws of inheritance to treat DNA variants as instrumental variables that mimic the randomization of individuals in clinical trials to two ‘treatment’ groups. Thus, genetic variants that have been associated with biomarkers or intermediate traits of interest (exposures) are then tested for association for disease outcomes (ideally in independent datasets) to infer a causal relationship. This approach has been successfully applied to numerous biomarkers, with the best results being related to classic cardiometabolic risk factors, such as elevated blood pressure, LDL, and triglycerides, that have been confirmed as causal drivers of atherosclerosis (Jansen et al., 2014). However, there are inherent limitations to MR that require careful consideration when evaluating causality of intermediate traits. For example, MR assumes that selected genetic instruments are valid and only associated with the biomarker being tested but not other related pathways and traits or confounding factors. In other words, the variants do not have pleiotropic effects. Second, variants may be weak genetic instruments for the exposure, which can lead to imprecise estimates and/or require larger sample sizes to boost power for detecting associations. Various statistical modifications to MR analyses have also been developed to address these limitations, such as two-sample MR (Hemani et al., 2018), multivariate MR (Sanderson et al., 2019), and TwoStepCisMR (Woolf et al., 2022). Thus, the large amount of publicly available summary statistics for numerous biomarkers and disease outcomes from published GWAS analyses renders MR an efficient and important analytical tool in systems genetic studies. One intriguing recent study developed an integrative MR framework to analyze gene-metabolite-phenotype associations from large-scale data (Auwerx et al., 2023). Application of this method uncovered new molecular links between omics datatypes, such as *SLC6A12* correlating with serum creatinine through modulation of the levels of the renal osmolyte betaine.

#### Colocalization

Colocalization approaches have become instrumental in deciphering the genetic basis of GWAS associations by integrating eQTL, meQTL, and pQTL data (Li and Ritchie, 2021). The premise of colocalization analysis is to determine if two associations, one to a molecular phenotype (such as gene expression) and the other to a complex trait or disease (i.e., GWAS), are driven by the same causal variants. Thus, colocalization approaches can identify candidate causal genes and prioritize functional variants that may be driving the observed associations. By leveraging molecular data reflecting different layers of biological regulation (e.g., gene expression, DNA methylation, and protein abundance), colocalization studies provide valuable insights into the potential biological mechanisms underlying complex traits and diseases. These approaches aid in translating GWAS findings by generating testable hypotheses (e.g., variant x influences the expression of gene y which in turn influences a complex trait) for experimental validation. In an elegant example of colocalization analysis, Al-Barghouthi and colleagues performed colocalization of tissue-wide eQTLs from GTEx and GWA significant loci for bone mineral density (BMD) (AlBarghouthi et al., 2022). These analyses uncovered >500 new candidate genes mediating variance in human BMD and validated PPP6R3 as a causal driver by ablating the gene in mice. In another study, Aberra and colleagues applied colocalization to multi-omic data to identify key genes and pathways which would distinguish BMI, WHR, and T2D (Aberra et al., 2023).

Several colocalization methods have been developed to facilitate the integration of molecular QTL data and GWAS, each with their strengths and limitations. Bayesian colocalization methods, such as COLOC (Giambartolomei et al., 2014), estimate the posterior probabilities of distinct colocalization hypotheses based on the observed summary statistics. Additional methods such as eCAVIAR (Hormozdiari et al., 2014) accommodate multiple causal variants. Most of the available methods do not require individual-level data, making them suitable for large-scale integrative analyses with publicly available summary statistics. Other approaches, such as summary-data-based Mendelian randomization (SMR) (Zhu et al., 2016) and joint likelihood mapping (Chun et al., 2017), rely on distinct statistical frameworks to assess colocalization. These methods have also been successfully applied to a wide range of complex traits and diseases, revealing novel candidate genes and functional variants.

#### Cross-tissue interactions

Systems genetics data from multiple tissues can be used to identify potential endocrine circuits mediating crosstalk. The method, first developed by Seldin et al., 2018, utilized correlation structure between the expression of individual genes in one tissue and all genes in a second tissue. If a given gene in the first tissue perturbs pathways in the second tissue, it may exhibit an overall correlation that rises above the noise of other gene correlations. The methods work best on animal models where environmental effects can be minimized, although it can also be applied to human datasets that are sufficiently large (Koplev et al., 2022). Recently, the method was used in mice to identify coagulation factor 11 as a novel mediator of liver-heart crosstalk with a significant impact on heart function and one form of heart failure (Cao et al., 2022).

#### Network modeling

One of the major premises of systems genetics is that biological components (transcripts, proteins, metabolites, etc.) do not act in isolation, but rather participate in complex networks that operate at the cellular, tissue, and organismal levels (Nadeau and Dudley, 2011; Civelek and Lusis, 2014). While reductionist approaches focus on linear events (e.g*.*, kinase phosphorylates protein), systems genetics seeks to understand how genetic variation alters network homeostasis and ultimately disease (AlBarghouthi and Farber, 2019). As a result, a key component of systems genetics studies is the reconstruction of cellular networks.

##### Co-expression networks

**Figure 2.**
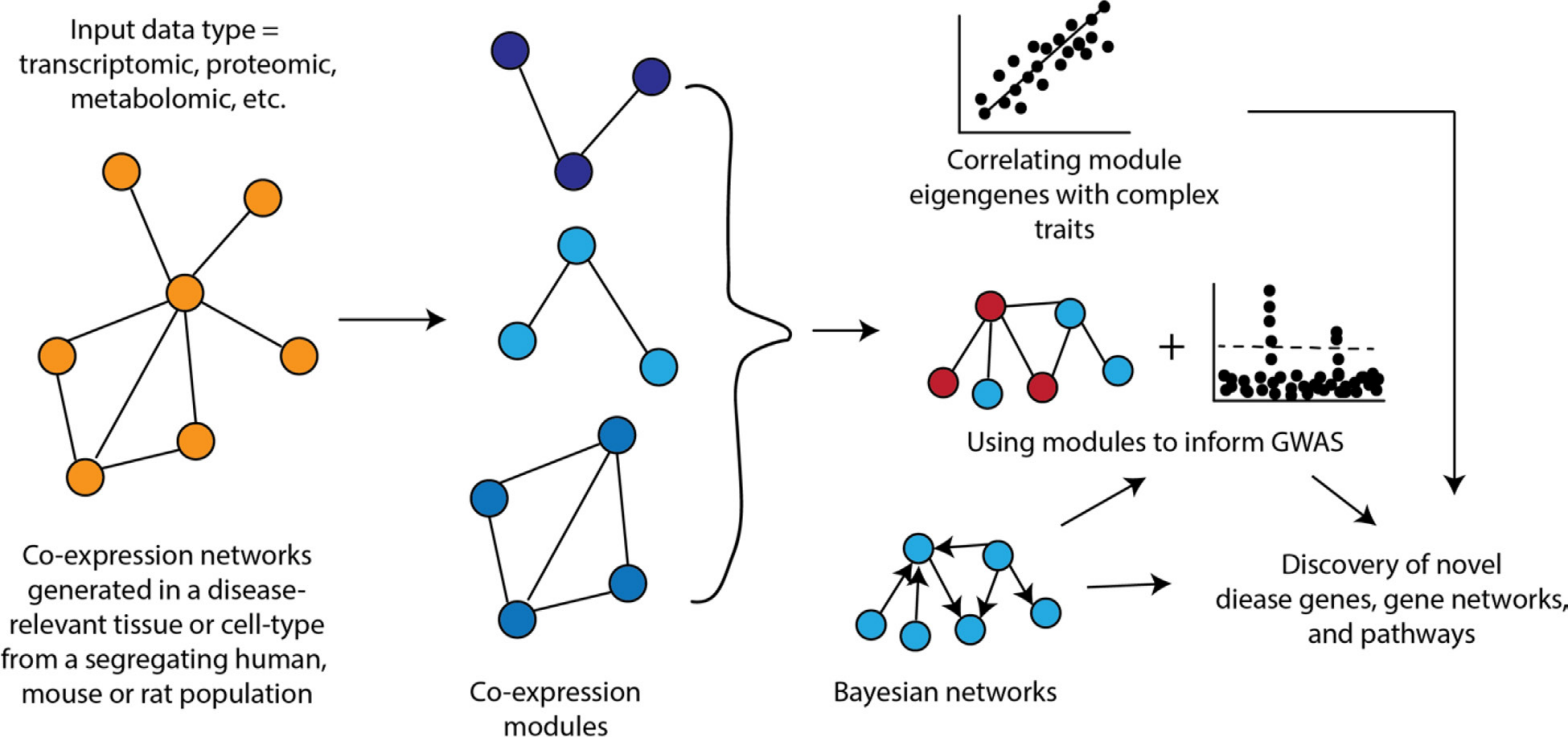
How networks can be used in systems genetics studies of disease.

Co-expression networks are one of the most popular types of networks used in systems genetics studies (Figure 2). In a co-expression network, nodes are genes and edges between genes represent a measure of the strength of their co-expression. Weighted gene co-expression network analysis (WGCNA) is a widely used tool for constructing co-expression networks (Langfelder and Horvath, 2008). WGCNA provides a way to organize biology by using the variation in gene expression generated by a series of perturbations (e.g., genetic background, treatments, etc.) to group genes into modules based on similarity in expression patterns. Studies across a wide range of species have demonstrated that genes whose expression co-varies often share similar functions and are members of the same biological pathway or process (McCarroll et al., 2004; Singer et al., 2005; Ala et al., 2008).

An important aspect of co-expression networks is that, compared to other biological networks, such as protein-protein interaction networks, they retain tissue or cell-type identity. Additionally, because these networks are generated across a set of samples, it is possible to relate the behavior of modules to other characteristics of the sample (Figure 1). For example, one of the first studies utilizing WGCNA identified liver tissue modules that correlated with differences in body weight in a mouse cross (Ghazalpour et al., 2006). Once modules are linked to complex traits, one can integrate the specific genes in the module for their involvement by integrating additional data (e.g., genetics data) or their place in the sub-network defined by the module, such as looking at module hub genes (Figure 1). Co-expression networks have been used to provide insight into a wide range of complex traits and diseases in rodents and humans such as chronic fatigue syndrome (Presson et al., 2008), CAD (McDermott-Roe et al., 2011), and diabetes (Keller et al., 2008).

Another use of co-expression networks has been to inform GWAS (Figure 1). The rationale behind this work is that causal genes from GWAS are often functionally similar and co-expression networks group genes with similar functions into modules. Therefore, it follows that using co-expression networks can help pinpoint potentially causal genes. The way some studies have implemented this strategy is to generate modules from disease-relevant tissues and then scan these modules to identify ones that are enriched for genes implicated by GWAS (located with associated loci) or for disease heritability. Pan et al. recently constructed a co-expression network from human adipose tissue and identified a module associated with waist-to-hip ratio adjusted for BMI (WHRadjBMI) (Pan et al., 2021). Additionally, regions around the 347 module genes were enriched for WHRadjBMI heritability. A transcription factor in this module was implicated as important in the context of WHRadjBMI through orthogonal data and its knockdown in human preadipocytes altered a large fraction of module genes (Pan et al., 2021). Calabrese et al. used a co-expression network generated from mouse bone to identify the ‘osteoblast functional module’ and use it to predict that *MARK3* regulated osteoblast activity and BMD (Calabrese et al., 2017), which was validated using an osteoblast-specific *Mark3* knockout mouse (Zhang et al., 2021b). In another recent example, Li et al. surveyed intestinal transcriptomics and cytokine abundances in 54 BXD strains fed a chow or HF diet (Li et al., 2023). The authors performed WGCNA on gene expression and applied association mapping to the module eigengenes to identify potentially causal drivers of intestinal bowel disease (IBD) phenotypes in mice. These modQTLs were intersected with IBD associations in UKBB, where MUC4 and EPHA6 were prioritized as key genes. Co-expression network principles have also been widely repurposed to evaluate molecular interactions beyond gene expression. For example, a recent *Caenorhabditis elegans* screen for argonaut protein localization was used to segregate members of the protein family into discrete modules which tracked with stress response and age (Seroussi et al., 2023).

##### Bayesian networks

A limitation of co-expression networks is that the direction of effect between genes is missing, and making definitive conclusions regarding causality is nearly impossible from a statistical perspective. Bayesian network reconstruction tools, on the other hand, have been developed to begin to address this limitation.

Bayesian networks are graphical models that represent probabilistic relationships among variables (Koller and Friedman, 2009). Bayesian networks have been widely used in various domains, such as artificial intelligence, machine learning, bioinformatics, and biology due to their ability to model complex relationships among variables and make inferences under uncertainty. They have also been widely used in the context of systems genetics studies. One of the first examples was the generation of Bayesian networks using transcriptomic data from segregating mouse populations to identify gene networks contributing to a wide range of metabolic phenotypes (Zhu et al., 2004; Mehrabian et al., 2005; Chen et al., 2008). These studies demonstrated the power of using networks to disentangle the molecular basis of complex traits.

Bayesian networks have been used in other ways in systems genetics studies (Figure 2). For example, Zhao et al., 2016, used tissue-specific Bayesian networks integrated with the results of GWAS for CAD to identify key driver genes for CAD. In this example, key driver genes were defined as those implicated by GWAS that were connected within a Bayesian network to known disease genes (Zhao et al., 2016). Their systems genetics framework identified tissue-specific relations among GWAS candidate genes and prioritized many genes as likely causal drivers of CAD. A similar approach was recently used to identify likely causal genes for osteoporosis (AlBarghouthi et al., 2021). In this analysis, the authors identified key drivers in a Bayesian network generated from mouse cortical bone transcriptomics data and integrated with GWAS data on BMD. A total of 66 high-priority likely causal genes for BMD were identified including *Glt8d2*, which was experimentally demonstrated to be involved in the regulation of BMD.

One particularly powerful Bayesian network approach that incorporates biological information (such as eQTL) is Mergeomics (Arneson et al., 2016; Shu et al., 2016), which builds on the explicit hypothesis that multi-omics modalities are functionally related and together can provide information on interconnected biological processes. Mergeomics uses only summary-level multi-omics data, which can be derived from different studies or even species. Briefly, multi-layer disease association signals are mapped to pathways or networks comprising interacting molecules to reveal pathogenic processes perturbed by individual omics variants as well as those affected by multiple omics layers. Recent applications of Mergeomics have yielded substantial insights into the tissue-specific biological processes and regulatory genes involved in individual diseases and those shared between diseases (Shu et al., 2017; von Scheidt et al., 2017; Chella Krishnan et al., 2018).

### Cross-species integration

While GWAS approaches have proven powerful in humans or model organisms, identification of conserved association signals at syntenic loci has not been widely observed. However, several notable successful examples of cross-species integration have been recently published. In one study, 60 loci were identified for ex vivo secretagogue-induced insulin secretion from isolated pancreatic islets of several hundred DO mice. Genes at these loci were significantly enriched for genes that localized to loci identified in human GWAS for diabetes-related traits (Keller et al., 2019). In a second study, integration of human lipid GWAS data with mouse liver co-expression networks from DO mice and the HMDP led to the identification of *SESN1* (Sestrin1) as a gene associated with cholesterol levels (Li et al., 2020). Prior to this study, Sestrin1 had not been known to be involved in lipid metabolism. More recent analyses have also demonstrated how incorporation of molecular networks can improve translation of GWAS results across species. For example, GWAS analyses in humans and rats identified hundreds of BMI-associated genes, of which 29 were found to overlap between the two species (Wright et al., 2023). However, by integrating molecular networks into the analyses, even greater convergence of GWAS signals was observed. Taken together, such studies highlight how cross-species systems genetics studies can identify novel candidate causal genes for complex traits and the need to develop additional computational tools to better carry out bi-directional translation of findings between humans and model organisms.

### Future directions

There will undoubtedly continue to be technical and computational innovations in multi-omics endeavors and this will speed progress in the systems genetics field. For example, above we discuss the value of incorporating long-read RNA-seq into systems genetics analyses (Glinos et al., 2022). A comprehensive view of common genetic variation of isoform-specific changes from these data has the potential to refine the definition of causal gene impacts compared to changes in expression alone from eQTL mapping. Further, there has been rapid progress in single-cell modalities. For example, recent technological advances have enabled widespread application of spatially resolved single-cell quantification (Moses and Pachter, 2022). While testing the robustness of these data in the context of common genetic variation presents significant challenges (discussed above with scRNA-seq), these measures expand our view of the regulatory landscape of single cells and their interactions with surrounding tissue. In addition, technological advances in methods such as proteomics and metabolomics continue to evolve. Lastly, artificial intelligence is already beginning to be applied to systems genetics data. Databases will continue to increase in size and number, and in silico analyses will become increasingly powerful.

Over the past decade, researchers have significantly refined mass-spectrometry-based methods to detect utilization of specific substrates using stable isotope tracing (Jang et al., 2018). Knowledge of these small molecule usages at a genetic scale presents substantial appeal in defining metabolism and associated disease mechanisms. In a recent study, Akingbesote and colleagues measured liver isotope flux, transcriptomics, and proteomics in five species (Akingbesote et al., 2023). They observed ~30,000-fold variation in the usage of substrates and suggested metabolic adaptations which differ between species, for example between mice and rats.

Systems genetics data also present a unique appeal to quantify complex interactions such as gene-by-diet responses (Nelson et al., 2022). Without measuring outcomes across diverse genetic backgrounds and environmental conditions, these interactions would be missed entirely which could prove relevant. In a recent example, Hodel and colleagues analyzed two longitudinal human patient cohorts for risk factors mediating coronary heart disease (Hodel et al., 2023). Here, the authors identified a unique interaction between polygenic risk of cardiovascular incidents and infection via *Fusobacterium nucleatum,* highlighting the complexities of GXE.

An important challenge will be to develop better methods for curating, storing, sharing, and integrating systems genetics data. We feel that a particularly useful goal would be to combine data from the various systems genetics groups into one searchable database that is accessible to biologists. One mechanism in which this type of database is implemented is available through the Alliance of Genome resources alliancegenome.org (Alliance of Genome Resources Consortium, 2022). This web-based API contains all data from seven model organism repositories (Saccharomyces Genome Database, WormBase, FlyBase, Mouse Genome Database, the Zebrafish Information Network, Rat Genome Database, and the Gene Ontology Resource for humans), which can be queried simultaneously at the level of genes, alleles, or disease models. Beyond centralizing these data and curating for streamlined comparisons, significant efforts will have to be applied to provide all researchers the ability to query and understand outputs from systems genetics analyses. This issue spans many areas of computational analyses related to biological data. Several efforts to centralize data analysis toolkits such as Docker, Github, and Jupyter have proved successful in this area; however, still require some knowledge of computational code. Undoubtedly, natural language processing will accelerate these improvements. For example, recent guidelines and considerations for utilization of chatGPT for coding optimization and generation have been summarized (Piccolo et al., 2023; Shue et al., 2023).

Major hurdles in applying unified analyses to common datatypes is differences in data structure, available pre-processing steps, and associated meta-data. Efforts have been proposed to reconcile these, such as the implementation of FAIR data principles (Wilkinson et al., 2016). These principles were first proposed in 2016 to suggest data standards, which maximize Findability, Accessibility, Interoperability, and Reusability. Given the utility of these principles in data management, these guidelines have been adopted by organizations such as the Research Data Alliance and been proposed as critical for the success of future data mining efforts such as machine learning (Scheffler et al., 2022). The approach should also be enhanced by the incorporation of additional data modalities, such as high-resolution clinical imaging data, single-cell gene expression data, and long non-coding RNA. In addition to quantitating the steady-state levels of macromolecules in populations, it may be possible to determine their rates of synthesis and degradation and how this is regulated.

One potentially fertile area of investigation is drug action using systems genetics data. An elegant example of this application was provided by Masson and colleagues who surveyed muscle proteomics from a diverse outbred population of mice exhibiting substantial variation in glucose metabolism profiles (Masson et al., 2023). The authors generated a pQTL footprint of proteins which co-mapped to MATSUDA index and linked these signatures to select compounds which were then subjected to two high-throughput drug screening platforms. Using these approaches, they identified thiostrepton as a compound which impacted glucose metabolism independent of the canonical insulin signaling pathway.

Systems genetics approaches are, of course, limited by that natural variation that is captured in the cohorts that are studied. In the case of human studies, certain ethnic groups have been disproportionately studied and, thus, some results may not apply the less studied groups. Clear racial, ethnic, and gender disparities have been described for most common diseases, emphasizing the need to incorporate more diverse populations in current research gold standards.
